# Non-Natural and Photo-Reactive Amino Acids as Biochemical Probes of Immune Function

**DOI:** 10.1371/journal.pone.0003938

**Published:** 2008-12-15

**Authors:** Marta Gómez-Nuñez, Kurtis J. Haro, Tao Dao, Deming Chau, Annie Won, Sindy Escobar-Alvarez, Victoriya Zakhaleva, Tatyana Korontsvit, David Y. Gin, David A. Scheinberg

**Affiliations:** 1 Molecular Pharmacology and Chemistry Program, Memorial Sloan-Kettering Cancer Center, New York, New York, United States of America; 2 Weill Cornell Graduate School of Medical Sciences, New York, New York, United States of America; Centre de Recherche Public-Santé, Luxembourg

## Abstract

Wilms tumor protein (WT1) is a transcription factor selectively overexpressed in leukemias and cancers; clinical trials are underway that use altered WT1 peptide sequences as vaccines. Here we report a strategy to study peptide-MHC interactions by incorporating non-natural and photo-reactive amino acids into the sequence of WT1 peptides. Thirteen WT1 peptides sequences were synthesized with chemically modified amino acids (via fluorination and photo-reactive group additions) at MHC and T cell receptor binding positions. Certain new non-natural peptide analogs could stabilize MHC class I molecules better than the native sequences and were also able to elicit specific T-cell responses and sometimes cytotoxicity to leukemia cells. Two photo-reactive peptides, also modified with a biotin handle for pull-down studies, formed covalent interactions with MHC molecules on live cells and provided kinetic data showing the rapid clearance of the peptide-MHC complex. Despite “infinite affinity” provided by the covalent peptide bonding to the MHC, immunogenicity was not enhanced by these peptides because the peptide presentation on the surface was dominated by catabolism of the complex and only a small percentage of peptide molecules covalently bound to the MHC molecules. This study shows that non-natural amino acids can be successfully incorporated into T cell epitopes to provide novel immunological, biochemical and kinetic information.

## Introduction

Specific T cell mediated immune responses involve T-lymphocytes that respond to linear peptide epitopes, typically between 8 and 20 amino acids in length. The peptides recognized by CD8+ T cells are 8–10 amino acids in length and are presented by class I major histocompatibility complex (MHC) molecules on the target cells. Class I MHC molecules have an affinity preference for peptides with particular major anchor residues, usually at amino acid positions 2 and 9. The stability of the peptide/MHC (pMHC) complexes correlates generally with the strength of the T cell response to the epitope.

Most cancer antigens are “self-antigens” expressed on normal cells and sometimes overexpressed on cancer cells. Immunogenic peptides derived from these tumor-associated proteins have been used in therapeutic vaccination protocols. Advances in the understanding of the cellular immune response to peptide antigens and structural studies of the pMHC have led to different strategies for improving cancer vaccines. One frequently studied antigen is the Wilms tumor protein (WT1), a zinc-finger transcription factor expressed during normal ontogenesis [1], [2], [3]. In adults, WT1 expression is limited to low levels in the nuclei of normal CD34+ hematopoietic stem cells, myoepithelial progenitor cells, renal podocytes and some cells in the testis and ovaries [4], [5], [6]. The WT1 gene product is over-expressed in hematological [7], [8], [9] and solid malignancies [10], making it an attractive target for immunotherapy. Short peptides derived from WT1 protein have been identified that generate a WT1-specific cytotoxic response [11], [12], [13], [14], [15], [16].

The feasibility of targeting WT1 has resulted in different clinical trials using four peptides with different adjuvants in the context of HLA-A0201 and HLA-A2402. Several trials are currently testing the WT1 235–243 natural sequence and modified sequence [17], [18], [19] in patients who are HLA-A2402; another set of studies tested the WT1 126–134 natural sequence [20] and modified peptides in patients who are HLA-A0201 [21].

Overcoming the often weak immunogenicity of and tolerance to tumor antigens may require appropriate modifications in the peptide sequence to increase pMHC or the T cell receptor (TcR) TcR-pMHC interactions; such changes should induce a more robust immune response if cross-reactivity to the native sequence was produced. One strategy used by our group and others is the design of synthetic analog peptides with natural amino acid substitutions at the anchor positions in the MHC molecule [18], [19], [21] that increase affinity, stimulate greater T cell recognition or break tolerance. Here we propose a new strategy to incorporate *non-natural amino acids* and *photo-reactive amino acids* into the CD8 peptide antigen. Incorporation of photo-reactive amino acids into the peptide sequences may provide unique opportunities to manipulate peptide-MHC binding interactions. The added modification of a biotin handle on the peptide allows kinetic studies of the peptide-MHC complex. Such non-natural amino acids could also serve to improve affinity at the pMHC or TcR recognition sites, break tolerance, or reduce catabolism of the peptide. In the present study, the incorporated non-natural amino acids into the sequence of WT1 peptides allowed us to determine if these alterations would increase avidity of binding to MHC, improve immunogenicity and result in cross-reactivity and cytolytic activity against WT1 expressing cancer cells, and study the MHC-peptide complex.

## Materials and Methods

### Peptides

**Figure 1:**
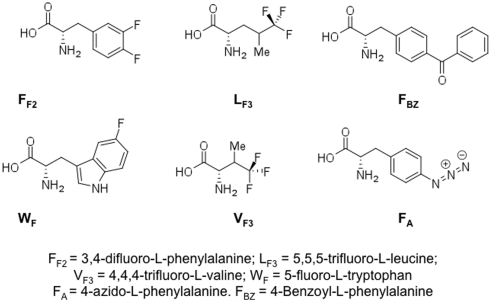
Chemical structures of the non-natural amino acids used in this study.

Potentially immunogenic peptides were selected from the Wilms' tumor (WT1) protein [21] (Table 1). Structures of the non-natural amino acids are shown in Figure 1. Peptides WT1 B, WT1B-S1Y, WT1B-S1V, WT1 B 8mer, WT1B-L2L_F3_, WT1 J, WT1J-C1Y and WT1J-M2Y were synthesized by Genemed Synthesis Inc. (CA, USA) using fluorenylmethoxycarbonyl chemistry, solid phase synthesis and purified by high-pressure liquid chromatography. The quality of the peptides was assessed by high-performance liquid chromatography analysis, and the expected molecular weight was observed using matrix-assisted laser desorption mass spectrometry. Peptides were sterile and greater than 70% pure. The peptides were dissolved in DMSO and diluted in phosphate-buffered saline (PBS; pH 7.4) or saline to give a concentration of 5 mg/ml and were stored at −80°C.

**Table 1:** Name and sequence* of the peptides.

Classification Of peptide	Peptide name: “B” series	Sequence: “B” series	Peptide name: “J” series	Sequence: “J” series
Native peptides	WT1B	SLGEQQYSV	WT1 J	CMTWNQMNL
	WT1B 8mer	LGEQQYSV	WT1J 8mer	MTWNQMNL
Heteroclitic peptides	WT1B-S1Y	YLGEQQYSV	WT1J-C1Y	YMTWNQMNL
	WT1B-S1V	VLGEQQYSV	WT1J-M2Y	CYTWNQMNL
Unnatural and photo-reactive peptides	WT1B-S1F_A_	F_A_LGEQQYSV	WT1J-C1F_A_	F_A_MTWNQMNL
	WT1B-S1F_A_-bio WT1B-S1F_bz_-bio WT1B-L2 L_F3_	F_A_LGEQ^bio^QYSV F_bz_LGEQ^bio^QYSV SL_F3_GEQQYSV	WT1J-C1F_bz_	F_bz_MTWNQMNL
	WT1B-S1V_F3_	V_F3_LGEQQYSV	WT1J-W4W_F_	CMTW_F_NQMNL
	WT1B-S1F_F2_	F_F2_LGEQQYSV		

* F_A_ = 4-azido-L-phenylalanine; L_F3_ = 5,5,5-trifluoro-DL-leucine; V_F3_ = 4,4,4-trifluoro-DL-valine; F_F2_ = 3,4-difluoro-L-phenylalanine; W_F_ = 5-fluoro-DL-tryptophan; bio = biotin; F_bz_ = 4-benzoyl-phenylalanine.

We synthesized the peptides: WT1B-S1V_F3_, WT1B-S1F_F2_, WT1B-S1F_A_-bio, WT1B-L2F_F3_,WT1B-S1F_bz_-bio, WT1J–W4W_F_, WT1J-C1F_bz_ (Table 1) using solid phase synthesis, on a Wang resin via automated synthesizer (PS3™ Peptide Synthesizer, Protein Technologies, Inc) using a single-coupling protocol. After cleavage from the resin using Reagent R (TFA∶thioanisole∶EDT∶anisole, 90∶5∶3∶2), the peptides were purified by high-pressure liquid chromatography (HPLC) on a reverse-phase C18 column (ZORBAX 300SB-C189.4 mm×25 cm, Agilent Technologies) with a 0 to 98% gradient of acetonitrile containing 0.1% trifluoroacetic acid (TFA). Peptide identity was verified by mass spectrometry. Biotinylation was achieved by using a commercially available Fmoc-L-glutamine amino acid containing biotin by way of a polyethylene glycol linker (Calbiochem) during peptide synthesis. Purities were greater than 90%.

Peptides WT1B-S1F_A_, and WT1J-C1F_A_ (Table 1) were synthesized by coupling the WT1B and WT1J 8-mers LGEQQYSV and MTWNQMNL, respectively, to the F-moc-4-azido-L-phenylalanine, using 0.4 M NMM/DMF as a coupling solution. After removal of F-moc by adding 20% piperidine/DMF, final deprotection and cleavage from the resin was done using Reagent R. Peptide identity was verified by mass spectrometry.

### Cell lines

TAP-deficient T2 cells were obtained from the American Type Culture Collection (Rockville, MD). 697 CML cells (WT1+) and SKLy-16 lymphoma cells (WT1-) were obtained from Sloan-Kettering stocks. Cells were grown in RPMI 1640 medium supplemented with 5% fetal bovine serum, 2 mM L-glutamine, β-mercaptoethanol, penicillin (100 U/ml) and streptomycin (100 µg/ml) in a humidified atmosphere containing 5% CO_2_ in an incubator.

### Peptide binding assay

Peptide binding to HLA-A0201 was assessed using a flow cytometry-based MHC stabilization assay [22], on the T2 cell line, which has HLA-A0201 expression and is TAP deficient in transport associated with antigen processing. In this assay, MHC binding of peptide in vitro was measured as the ability of exogenously added peptides to stabilize the class I MHC/ β_2_-microglobulin structure on the surface of the T2 cell line. Briefly, T2 cells were incubated in 24-well flat bottom plates at 5×10^5^ cells/well in a 600 µl volume of serum-free medium with human β2-microglobulin at a final concentration of 10 µg/ml with and without peptides at concentrations between 50 and 1 µg/ml for 16 h at 37°C. Cells were then incubated with 5 µg/ml brefeldin A (Sigma) for 2 h at 37°C. After washing twice with FACS buffer (2% human serum in PBS), cells were incubated for 30 minutes in the dark at 4°C with FITC labeled anti-human HLA-A2 antibody, (BD Pharmingen ™). Fluorescence was determined using a Cytomics ™ FC 500, (Beckman Coulter) and analyzed using the FlowJo program. Assays were run with a positive reference peptide of high affinity (a decapeptide from the antigen S of the hepatitis B virus, FLPSDYFPSV). For clarity, the reference binding curves are not shown in the panel. Each concentration of peptide (1, 10 and 50 µg/ml) was assayed in triplicate wells. For the time course experiments, 50 µg/ml of peptide was used and cells were incubated for various times up to 24 hr before flow cytometric assay to follow the loss of the peptide MHC complex from the cell surface. Each experiment was done at least twice.

A different kinetic and quantitative assessment of peptide binding was conducted using another flow cytometry assay in which binding was measured using an anti-biotin mouse monoclonal antibody conjugated to AlexaFluor 488 (Invitrogen). In this assay, T2 cells were treated with indicated peptides for various times, exposed to ultraviolet light as described earlier, then washed twice with PBS containing 1% human serum. Then, in aliquots of 10^5^ cells in 100 µl volume of PBS containing 1% human serum, cells were treated with 2.5 µg/ml of the anti-biotin AlexFluor 488 conjugate for 20 minutes on ice in the dark. Cells were washed twice in PBS then analyzed by flow cytometry.

Other experiments were done in which acid stripping of peptides was used to measure residual peptides on the surface of MHC molecules. Following UV light treatment, T2 cells were washed twice with PBS containing 1% human serum. Cells were re-suspended in stripping buffer (0.13 M citric acid, 0.06 M sodium phosphate monobasic, pH 3.0, 1% BSA) for 120 seconds on ice, then five volumes of neutralizing buffer were added (0.15 M sodium phosphate monobasic, 1% BSA, pH 7.5) before pelleting cells for 10′ at 4 C at 1,200 rpm. Cells were then washed twice in PBS with 2% BSA before staining for flow cytometry.

For peptide internalization studies, T2 cells were treated with peptides as described above, then incubated for 2 hr at 37 degrees. The cells were split into two pools and one was left untreated and the other washed twice to remove unbound and non-internalized peptides. Both pools of cells were then incubated and tested for peptide bound to the cell surface at 4 and 22 hours.

### In vitro immunization and human T-cell cultures

After Institutional Review Board approved informed consent, peripheral blood mononuclear cells (PBMCs) from HLA-A0201 positive healthy donors were obtained by Ficoll-density centrifugation. Dr. Bo Dupont and Ms. Alice Yeh of the Immunology Program, Sloan-Kettering Institute generously provided the HLA genomic typing of the cells for this study.

Monocytes were isolated from the donors using magnetically isolated CD14+ (Milteny, CA, USA) positive fractions and used as antigen presentation cells (APCs) in the first stimulation. Dendritic cells were generated as follows: CD14 positive fractions were cultured in RPMI 1640 medium supplemented with 1% autologous plasma, previously heat-inactivated, 500 U/ml recombinant human interleukin IL-4 (R&D Systems, Minneapolis, MN, USA) and 1000 U/ml recombinant human granulocyte-macrophage colony-stimulating factor (GM-CSF) (Immunex, Seattle, WA, USA). After 2 and 4 days of incubation, part of the medium was exchanged for fresh culture medium supplemented with IL-4 and GM-CSF, and culture was continued. On day 6, half of the medium was exchanged for fresh medium and a maturation cocktail was added: IL-4, GM-CSF, 400 IU/ml IL-1β (R&D Systems), 1000 IU/ml IL-6 (R&D Systems), GM-CSF, 10 ng/ml TNF- alpha (R&D Systems) and 1 µg/ml pGE2 (Sigma, St. Louis, MO). On day 7, the cells were harvested and used as APCs for the second stimulation.

T lymphocytes were isolated from the CD14 negative fractions using a pan T cell isolation kit. Non T cells, i.e., B cells, NK cells, DCs, monocytes, granulocytes and erythrocytes were indirectly labeled by using a cocktail of biotin-conjugated antibodies against CD14, CD16, CD19, CD36, CD56, CD-123 and Glycophorin A, and anti-biotin microbeads MoAb (milteny CA, USA). CD8+ lymphocytes were isolated from the CD14 negative fractions using CD8 microbeads (Milteny, CA, USA). Purity was typically more than 98% on flow cytometry.

T lymphocytes (CD3+ or CD8+) were stimulated the first time at a 5∶1 effector∶target (E∶T) ratio with monocytes (CD14+), in RPMI 1640 medium supplemented with 5% heat-inactivated human autologous plasma with WT1 synthetic peptides at a concentration of 20 ug/ml and β2 microglobulin (Sigma, St Louis, MO, USA) at 5 ug/ml in 6-well plates in the presence of 10 ng/ml recombinant human IL-15 (R&D Systems). After 7 days T cells were restimulated using the dendritic cells generated the week before at a 30∶1 E∶T ratio, together with WT1 synthetic peptides, β2 microglobulin and IL-15 at the same concentrations used in the first stimulation. After culture for 2–3 days fresh medium with IL-15 was added. After the second stimulation T cells were stimulated weekly using CD14+ or DCs as a target (10∶1 or 30∶1 E∶T ratio respectively) depending on the availability of cells together with WT1 peptides, β2 microglobulin and IL-15 at the same concentrations. After the second and following stimulations, interferon (IFN)-γ secretion of these cells was then examined by ELISPOT.

### IFN-γ ELISPOT

HA-Multiscreen plates (Millipore, Burlington, MA, USA) were coated with 100 µl of mouse anti-human IFN-γ antibody (10 µg/ml); clone 1-D1K, Mabtech, Sweden) in PBS, incubated overnight at 4°C, washed with PBS to remove unbound antibody and blocked with RPMI/autologous plasma for 2 h at 37°C. Purified T cells (CD3+ or CD8+, more than 95% pure) were plated at a concentration of 1×10^5^/well. T cells were stimulated with 0.5×10^5^ T2 cells per well (2∶1 E∶T ratio), or 1×10^4^ CD14+ cells (10∶1 E∶T ratio) or 3.3×10^3^ DCs (30∶1 E∶T ratio) pulsed with 5 µg/ml of β_2_-microglobulin and various test peptides at 20 µg/ml. Negative control wells contained APCs with or without T cells or T cells alone plus irrelevant control peptide. Positive control wells contained T cells with APC plus 10 µg/ml PHA (Sigma). All conditions were done in triplicate. After incubation for 20 h at 37°C, plates were extensively washed with PBS/0.05% Tween and 100 µl/well biotinylated detection antibodies against human IFN-γ (2 µg/ml; clone 7-B6-1, Mabtech, Sweden) was added. Plates were incubated for an additional 2 h at 37°C and spot development was performed as described [23]. Spot numbers were automatically determined with the use of a computer-assisted video image analyzer with KS ELISPOT 4.0 software (Carl Zeiss Vision, Germany).

### Chromium-51 release assay

The presence of specific cytolytic T lymphocytes (CTLs) was measured in a standard 4 h. chromium release assay as described (22). Briefly, target cells were incubated with or without synthetic peptides at 50 µg/ml overnight in the presence of β_2_-microglobulin at 5 µg/ml, after which they were labeled with 100 µCi of Na_2_^51^CrO_4_ (NEN Life Science Products Inc., Boston, MA, USA) for 1 h at 37°C. After washing, target cells were resuspended in complete media at 3×10^4^/ml and plated in a 96-well U-bottom plate (Becton Dickinson NY) at 3×10^3^ cells/well with effecter cells at effecter to target (E/T) ratios ranging from 100∶1 to 10∶1. All conditions were performed in triplicate. Plates were incubated for 4 h at 37°C in 5% CO_2_. Supernatant fluids were harvested and radioactivity was measured in a gamma counter. Percent specific lysis was determined from the following formula: 100×((experimental release-spontaneous release)/(maximum release-spontaneous release)). Maximum release was determined by lysis of targets in 2.5% Triton X-100.

### MHC-peptide photo-activation, cross-linking, and western blot analysis

T2 cells were incubated overnight at 37°C in serum-free RPMI medium in the absence (negative control) or presence of 50 µg/ml of the photo-activatable peptides WT1B-S1F_A_–biotin and WT1B-S1F_BZ_–biotin. Following incubation, cells were exposed to a 40S-XX Sylvania 115 volt short wavelength UV bench lamp for 1 min at a range of 5 cm at room temperature (azido moiety) or a Rayonet 17/15W 120 V long wavelength UV box for 30 minutes on ice (for benzophenone moiety). For the time-course experiments, cells were incubated in 5% dialyzed FBS during peptide incubation and given 2× fresh media containing 5% dialyzed FBS in RPMI following UV exposure. Cells were then transferred to Eppendorf tubes and washed twice with phosphate-buffered saline (PBS). Cell pellets were lysed in 1 ml of RIPA buffer containing protease inhibitor cocktail (Roche) and incubated with 7 ul of 50% slurry streptavidin bead solution (Pierce) for 1 hr at 4°C, then washed 4 times with PBS supplemented with 0.1% SDS. The streptavidin-protein complexes were boiled in 2 times SDS sample buffer for 5 minutes (BioRad), resolved by SDS-PAGE, transferred to a PVDF membrane, and blocked in Tris buffered saline (TBS) containing 5% milk. The membrane was probed with a 1∶250 dilution of an anti-HLA-A goat polyclonal antibody (Santa Cruz, A-18) overnight at 4°C, washed twice with TBS containing 0.1% Tween-20 (TBST), probed with an HRP-conjugated donkey anti-goat IgG (Santa Cruz Biotechnology) at 1∶5000 dilution in TBS with 5% milk for 2 hours at room temperature, then developed using a chemiluminescent substrate (Pierce).

## Results

### Selection of WT1 peptides and non-natural amino acids

We selected two native nonapeptides from the WT1 protein (Table 1), WT1 J (235–243) and WT1 B (187–195) because we and others have shown their ability to generate a T cell response, either to the native sequence or to modified synthetic analog peptides [16], [17], [18], [19], [21]. We focused generally on changes to amino acids in position one as previous work [21] showed that these changes had a large impact on MHC binding when anchor residues were already present. Here we first employed non-natural amino acids in an attempt to improve MHC binding and immunogenicity. Fluorinated amino acids were of special interest because fluorine and hydrogen are nearly isosteric and fluorination generally causes only modest structural perturbation compared to hydrocarbon analogues; however fluorocarbons have elevated hydrophobicity. In general, canonical medium to high affinity anchor motif peptides for MHC class I make use of long hydrophobic amino acids to bind to the MHC anchor pockets, so we hypothesized that increasing the hydrophobicity in the area of the anchor residues could induce better binding and immunogenicity [21]. The following fluorinated peptides were synthesized (Table 1, Fig. 1): WT1B-L2L_F3_, WT1B-S1V_F3_, WT1B-S1F_F2_ and WT1J-W4W_F_. In contrast with the other non-natural amino acid sequences, which were modified in or near the position 2 anchor residue, WT1J-W4W_F_ has the fluorinated amino acid substitution in position 4, which is not an anchor residue, because hydrophobic structural elements on central regions may play a critical role in eliciting CTL responses [24].

The non-natural azido-phenylalanine was of interest because phenylalanine is a long hydrophobic amino acid that could induce a better binding to the MHC anchor. The incorporation of azido amino acids was an attractive modification because the azide group has the ability to survive cellular metabolism [25] and is photo-reactive. We hypothesized that photo-activation of the azido group could be used to covalently lock the peptide into amino acids present in the MHC pocket, thus providing “infinite affinity” at the site. In this way, use of azido amino acids may provide unique opportunities to manipulate peptide-MHC binding interactions to study the effect of permanent occupation of a peptide in the MHC binding site and also to allow kinetic studies of the peptide-MHC complex by use of a biotin handle. The following azido-peptides were synthesized (Table 1, Fig. 1): WT1B-S1F_A_ and WT1J-C1F_A_. We also prepared a second class of photo-reactive peptides with a less labile reactive moiety, benzophenone (WT1J-C1F_bz_ and WT1B-F_BZ_), for the same purpose. 8-mers (WT1B 8mer and WT1J 8mer), peptides lacking the first amino acid were also included, in order to compare their MHC binding affinity to the several other analogs with changes at position 1.

### Binding of non-natural peptides to HLA-A0201 molecules

Peptide binding to HLA molecules is a prerequisite for peptide presentation and T-cell recognition. As computer algorithm binding prediction programs include only natural amino acids, and are relatively inaccurate [22] we directly measured the interaction between the non-natural peptides and the HLA-A0201 molecules using the T2 binding and stabilization assay (supplemental figures S1 and S2). A dose-response curve was done to get measurements of relative avidity among peptides and a time course of stabilization was done as this may better reflect immunogenicity of the peptides.

#### Analogs of WT1 J

The non-natural analogs of WT1J were analyzed in a time-course binding assay and a peptide concentration dose-response assay. WT1J-C1F_A_ bound well, in its unreactive form and after UV irradiation activation as well. As expected, the control 8-mer peptide lacking the first amino acid showed no significant binding. In each experiment, an HBV peptide with known high avidity was used as an internal control of assay integrity. The fluorinated WT1J-W4W_F_ showed the best binding profile in both a time- and a dose-dependent manner compared with WT1J and the rest of the J analogs. This was surprising because the modification was not near an anchor residue.

#### Analogs of WT1 B

Three out of the four non-natural analogs improved the measured binding to the HLA-A0201 molecules, compared to the native WT1B peptide. WT1B-S1Y, a natural synthetic analog and the tri-fluoro WT1B-S1VF_3_ generally showed the best binding profiles. The control WT1B 8mer peptide showed weak binding. The photo-activatable analog of WT1B showed similar behavior to the photo-reactive WT1J analog. After irradiating with UV light, WT1B-S1F_A_ showed slightly lower binding ability, which may have been due to hydrolysis and loss of the peptides after irradiation. The more stable photoreactive WT1B-F_bz_-bio showed similar binding to both the natural WT1B sequence and the synthetic analog WT1B-S1Y. In all these experiments, the HBV peptide was used as an assay integrity positive control and showed a binding index of 5–7 at the highest concentrations tested (data not shown).

### Photo-activatable peptides bind to HLA-A0201 MHC molecules

**Figure 2:**
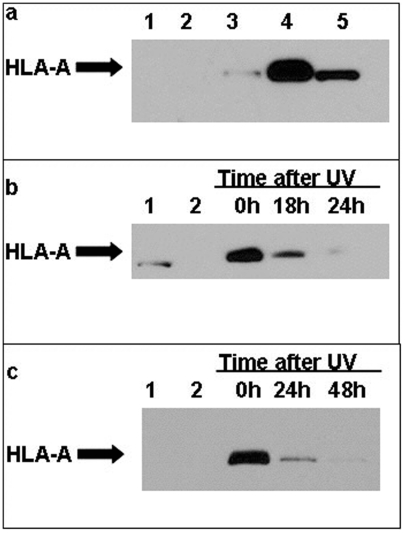
HLA western blot following biotin-peptide pull-down. Panel a. T2 or control cells were treated with reagents indicated below overnight at 37°C and then were exposed or were not exposed to UV light. Lysates were then resolved on SDS gels and blotted for the presence of HLA-A. Lane 1: WT1B-S1F_A_ in control HLA-A0201-negative cells (KG-1); Lane 2: T2 cells without peptide; Lane 3: T2 cells and WT1B-S1F_A_ without UV irradiation; Lane 4: T2 cells and WT1B-S1F_A_ plus 1 min short wavelength UV irradiation; Lane 5: Positive control cells whole lysate with antibody. Panel b. Following photo-activation of peptide, T2 cells were incubated for the times indicated on the gel, prior to lysis and western blot analysis. Lane 1: Positive control cells with whole lysate and antibody; Lane 2: T2 cells without peptide. Lanes 3–5: T2 cells and WT1B-S1F_A_ plus 1 min short wavelength UV incubated for 0, 18, and 24 hr before analysis. Panel c. Conducted as in panel ‘b’ but using the WT1B-S1F_bz_ peptide. Lane 1: T2 cells without peptide; Lane 2: WT1B-S1F_bz_ with no UVirradiation; Lanes 3–5: T2 cells and WT1B- S1F_bz_ plus 30 min long wavelength UV followed by incubation for 0, 24, and 48 hr before analysis.

To demonstrate the ability of photo-reactive peptides to bind covalently to HLA molecules in live cells, we determined whether MHC molecules could be pulled down and identified in lysed cells after incubation with the biotinylated peptides and UV irradiation. T2 cells or KG-1 (negative control cells lacking the HLA-A0201 allele) were incubated with WT1B-S1F_A_-biotin photo active peptide or media alone overnight to non-covalently bind empty MHC class I molecules on the T2 cell surface. At 20 h, the peptides were photo-activated to allow cross-linking to their target proteins, then complexes were isolated and resolved on SDS-PAGE and developed using anti-HLA western blot (Fig. 2).

Specific covalent association of the activated azido-peptide with the HLA-A0201 on the cells was demonstrated (Fig. 2A, lane 4.) HLA-A0201 negative control cells with peptide (lane 1) and HLA-A0201 positive cells not exposed to peptide (lane 2) did not display the HLA-A molecule upon western blot analysis. T2 cells treated with WT1B-S1F_A_ peptide, but not exposed directly to UV light demonstrated a small amount of MHC, (lane 3) consistent with its high avidity for the target. Ponceau stains showed no significant other bands present. These results demonstrated that derivatives of the WT1B sequence harboring photo-activatable residues are capable of covalently binding HLA-A molecules on live cells following exposure to UV light.

To determine the kinetics of the maintenance of the interaction of the peptide-MHC complex, we repeated the experiment of adding the photo-reactive peptide to the T2 cells followed by UV exposure. Next, cells were incubated for 0, 18 and 24 h at 37°C to allow kinetic analysis of the complex (Fig. 2B). The peptide-MHC complex was almost completely lost after 24 hours of incubation, presumably due to catabolism of the peptide structure, as cleavage of the biotin from the amino acid is unlikely.

Similarly, the other WT1B peptide derivative harboring a benzophenone moiety rather than an aryl azide (WT1B-F_bz_), also demonstrated covalent binding to HLA following UV-induced cross linking (Fig. 2C). This peptide retained a small portion of its binding after 24 hours, but was nearly all lost after 48 hours, suggesting a more robust cross-linking from the benzophenone-containing peptide versus the azido-containing peptide (compare panel 2B to 2C). Cells treated with WT1B-F_BZ_ , but not exposed to UV light did not demonstrate cross-linking of HLA-A molecules. These data probably can be attributed to the greater inherent stability of benzophenone moiety versus that of aryl azides in the presence of background light [26]. These kinetics are in agreement with the T2 HLA stabilization assay, which demonstrate that following 24 h of incubation of cells at 37°C, the expression of the peptide-MHC complex on the cell surface is significantly reduced (supplemental figure S1).

### Induction of a peptide specific T cell response as detected by IFN-γ assay

Purified CD8+ T cells from healthy donors were stimulated with monocyte-DC antigen presenting cells pulsed with the amino acid sequences containing non-natural amino acid analogs to test their ability to generate peptide-specific CTLs. After two or three T cell stimulations *in vitro*, an IFN-γ ELISPOT assay was performed to measure specific and cross-reactive responses.

#### Analogs of WT1 J

**Figure 3:**
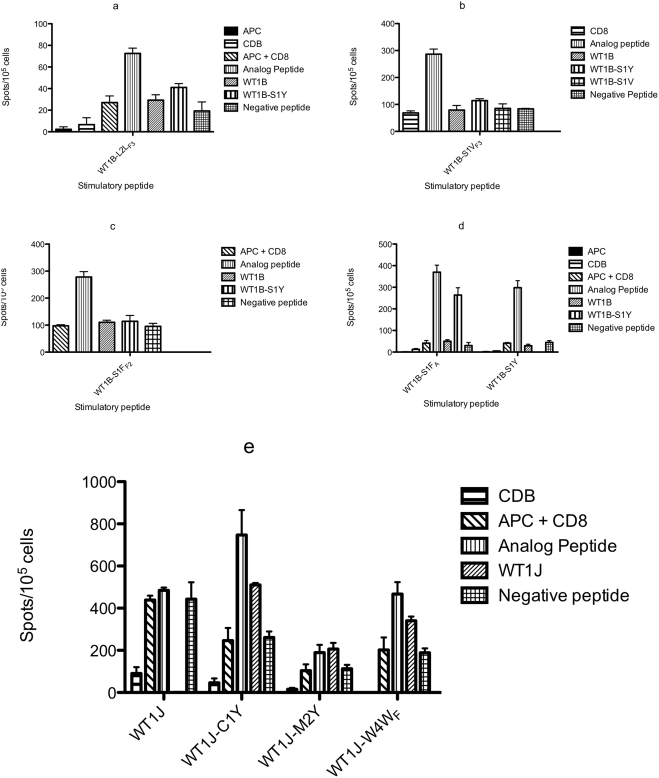
CD8+ IFN-γ ELISPOT from an HLA-A0201 donor. The assay was conducted as described in Materials and Methods. T cells were stimulated *in vitro* with the WT1J peptides shown in the *X* axis and then challenged with the peptides shown in the legend. The *Y* axis represents the number of spots per 1×10^5^ CD8+ T cells.

WT1J is a weak immunogen [21]. The non-natural analogs of WT1 J were tested *in vitro* against HLA-A0201 donors. We compared the WT1J native peptide with the natural peptide analogs, WT1J-C1Y and WT1J-M2Y, and with the non-natural peptide WT1J-W4W_F_. Under these conditions, all three analog peptides generated T cell responses to themselves above background, whereas the native J peptide did not (Fig. 3). Importantly, the T cells generated in the presence of both the natural heteroclitic analogs and the non-natural analog, WT1J-W4W_F_, were able to recognize to varying degrees, the native sequence of WT1J in the setting of HLA-A0201 class I molecules. After three rounds of stimulations, WT1J-C1Y and WT1J-W4W_F_ were able to generate a robust immune response. We also tested the immunogenicity of the photo-activatable WT1J-C1F_bz_ and WT1J-C1F_A_ peptides. Both showed a weak immune response that was even lower after UV irradiation (data not shown). These data are consistent with the T2 binding data.

#### Analogs of WT1 B

All of the peptides containing non-natural amino acids (WT1B-L2L_F3_, WT1B-S1V_F3_, WT1B-S1F_F2_ and WT1B-S1F_A_) were able to generate specific-CTL that recognized the immunizing peptide (Fig. 3). WT1B-S1F_A_ also showed cross-reactivity with the analog WT1B-S1Y, but none of the T cells stimulated by any of the B series analogs showed cross-reactivity with the native WT1B sequence. WT1B-S1F_A_ demonstrated a comparable response to WT1B-S1Y, the natural heteroclitic analog that has been described previously as a more immunogenic peptide compared with the native sequence WT1B [21].

### Quantitative and kinetic analysis of peptide cross-linking

Although western blot analysis indicated that the photo-activatable peptides WT1B-S1F_BZ_-bio and WT1B-S1F_A_-bio covalently bound HLA molecules, photoactivation of these peptides did not stimulate cytotoxic T cells (data not shown). To further assess the reasons for this, we treated T2 cells with the WT1B-S1F_BZ_-bio photo-activatable peptide for 16 hours then exposed them to UV light and measured the amount of peptide bound on the cell surface using an anti-biotin monoclonal antibody conjugated to an AlexFluor 488 fluorophore. Exposure to UV light did not significantly increase the amount of peptide bound on the cell surface (Table 2A). Binding was specific for the test peptide as compared to a control peptide containing just five residues, and also harboring a benzophenone moiety and a biotin handle.

**Table 2:** Peptide Binding Photoactivation and Kinetic studies.

A: Peptide binding following photoactivation	
Sample	Mean fluorescence^a^
0 uM WT1B-S1Fbz	1.54
0 uM WT1B-S1Fbz+UV	2.68
1 uM Control Peptide	1.56
1 uM Control Peptide+UV	2.76
1 uM WT1B-S1Fbz	41.5
1 uM WT1B-S1Fbz+UV	43.2

B: Peptide binding following acid stripping	
Sample	Mean fluorescence
0 uM WT1B-S1Fbz	1.34
0 uM WT1B-S1Fbz+UV	2.62
1 uM Control Peptide	1.47
1 uM Control Peptide+UV	2.12
1 uM WT1B-S1Fbz	1.63
1 uM WT1B-S1Fbz+UV	2.59

C: Peptide Binding is not affected by Internalization	
Sample	Mean fluorescence
0 uM WT1B-S1Fbz	2.02
1 uM WT1B-S1Fbz 2 h	8.74
1 uM WT1B-S1Fbz 6 h	44.4
1 uM WT1B-S1Fbz 6 h+wash	6.69
1 uM WT1B-S1Fbz 24 h	23.4
1 uM WT1B-S1Fbz 24 h+wash	2.16

a mean fluorescence data were similar to median fluorescence data.

This experiment was repeated with an additional acid-stripping step to remove non-covalent peptide-HLA interactions following exposure to UV light (Table 2B). The data confirmed that the number of peptides cross-linked to HLA molecules was small.

We then performed a pulse-chase experiment in which we treated cells for 2 hours with peptide, washed cells of unbound or non-internalized peptide, and then followed the amount of peptide bound to the surface after incubation for an additional 4 or 22 hours. Total peptide on the surface continued to fall over time after washing, to background levels at the 22 hour time point, suggesting that either the peptides were not being internalized prior to binding to HLA molecules or they were not being re-circulated and presented in HLA molecules on the surface after internalization (Table 2C). Together, these data show that, in addition to rapid catabolism of peptide-HLA complexes shown in Figure 2, the low efficacy of cross-linking by the photo-activatable peptides may hinder the production of adequate numbers of “infinite” affinity peptide-HLA complexes to generate potent cytotoxic responses.

### Peptides containing non-natural amino acids stimulate cytolytic T cells

**Figure 4:**
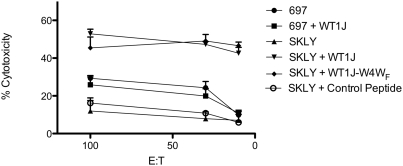
Cytotoxicity assay with CD8 T cells from an HLA-A0201 donor after three stimulations *in vitro*. The assay was conducted as described in Materials and Methods. The *X* axis shows different ratios between T cells to target cells. The targets and the peptides used to pulse them are shown in the legend. The *Y* axis shows percentage of cytotoxicity.

WT1J-C1Y is the natural heteroclitic analog that has been described previously as a more immunogenic peptide compared with the native sequence WT1J. Despite its high affinity for the MHC and its ability to generate positive and cross-reactive ELISPOT data, WT1J-C1Y has not shown cytotoxic activity against WT1 expressing target cells except when pulsed with the specific peptide [21]. We tested the CD8 T-cells after the third stimulation with the non-natural peptide WT1J-W4W_F_ in a chromium-51 release assay using WT1 expressing cells and peptide-pulsed target cell lines. CD8+ T cells generated *in vitro* from HLA-A0201 donors were able to kill the 697 cell line (HLA-A201+, WT1+), whether pulsed or not pulsed with the native WT1J peptide (Figure 4). In addition, these CD8+ T cells did not recognize SKLY16 cells (HLA-A201+, WT1-), unless they had been pulsed with the native WT1J peptide or the non-natural peptide WT1J-W4W_F_ showing that this was a specific peptide restricted response.

## Discussion

Protein engineering by amino acid substitution has usually been restricted to the 20 naturally occurring amino acids. Recently, the incorporation of non-natural amino acids into proteins in living cells has greatly expanded the types of amino acids available for different applications in protein engineering and functional studies [27], [28]. Non-natural amino acids also have been introduced into peptide-based vaccines to enhance catabolic stability because natural antigenic peptides have short bioavailability as a consequence of high susceptibility to serum or tissue proteases. Altered sequences may also promote greater immunogenicity of the peptide by providing higher affinity to MHC binding pocket anchor residues or by breaking immune tolerance to the native amino acid sequence, so long as cross-reactivity to the original protein sequence can be preserved.

Our study shows that an amplified T cell response can be achieved sometimes by use of non-natural amino acids in the sequence of WT1 peptides. The peptides synthesized could elicit an effective T cell response to themselves and were capable of sometimes stimulating T-cell responses stronger than those of their original native peptides. Moreover, T cells stimulated with the peptide WT1J-W4WF were able to recognize the native WT1J sequence and showed cytotoxicity against an HLA-A0201, WT1+ acute leukemia cell line. Whether the enhanced response is derived from the location of the modification or its biochemical nature cannot be determined from these studies.

In addition, we were able to construct peptides that had photo-affinity labels incorporated into them, which allowed the peptides to be covalently attached to the MHC molecules after binding. These peptides demonstrated kinetic data consistent with turnover of the peptide-MHC complex over 24 hours, a time comparable to the turnover of high avidity peptides not bound covalently to MHC. The covalently bound peptides, despite their “infinite” affinity for MHC, were not demonstrably more immunogenic, which suggests that once a peptide has achieved sufficient affinity for the MHC molecule, further improvements in immunogenicity will be limited first by the natural catabolism of the complex and ultimately by the recognition and cross-reactivity of the T cell. Furthermore, the data indicated that a very small fraction of the photoactive peptide was covalently bound. While few peptides need to be bound to MHC generate T cell responses, it is possible that a higher efficiency of labeling might yield a stronger response, despite the catabolism noted.

There have been other reports of improvements of infectious disease vaccine epitopes by altering the peptides with non-natural amino acid substitutions, but the present work is the first to show stimulation by such sequences of cross-reactive, cytolytic T cell responses to a cancer self-antigen. Studies of anti-viral immunity using an Epstein-Barr virus subdominant epitope derived from the membrane protein LMP2 that represents the target of HLA-A0201 restricted CTL responses, was improved by preparing an analog carrying one amino acid substitution at a non-anchor position that was highly sensitive to proteolysis. Three peptides with higher enzymatic resistance were found that stimulate CTL responses against the natural epitope [29]. In order to improve the same epitope analogs containing *cis*- and/or *trans*-4-aminocyclohexanecarboxylic acid (ACCA) replacing dipeptide units were made. All peptides showed higher enzymatic resistance and some *trans*-ACCA derivatives also stimulated CTL responses against the natural epitope [30]. A biologically active peptide containing beta-amino acids was also synthesized. Oligomers of 3-hydroxybutanoate and/or beta-homoalanine were incorporated in the central part of HLA-B27 epitope of HIV gp 120, without impairing MHC binding [31].

Other groups have replaced residues that are not essential for MHC binding by non-natural amino acids that are not recognized by T-cells, or that might be able to antagonize autoreactive T cell clones in autoimmune diseases. In this setting, *Krebs et al.* synthesized three peptides with aromatic amino acids (alfa-naphthylalanine, beta-naphthylalanine or homophenylalanine) in the middle part of a HLA-B27 restricted epitope enhancing the binding of the peptide to their host MHC protein [32]. Recently in an effort to increase the affinity of peptides for HLA-B2705 *Jones et al.* synthesized two modified epitopes with a non-natural arginine analogue at position 2. The modified peptides have decreased ability to bind the HLA-B2705 molecules and were not recognized by CD8+ T cells [33].

A few studies have incorporated non-natural amino acids into T cell epitopes for tumor vaccines. *Webb et al.* engineered an HLA-A2 restricted NY-ESO1 epitope, a tumor antigen of the cancer-testis family, with modification of the C-terminal cysteine residue to 2-aminoisobutyric acid, a cysteine iso-stereomer, that prevented the dimerization resulting from oxidation of this residue, and this modification did not affect HLA-A2 peptide stability, nor CTL recognition [34]. The same group incorporated beta-amino acids into peptides to decrease proteolysis [35]. *Guichard et al.* also synthesized a melanoma MART-1_27–35_ analogue with beta-amino acid at the putative TcR contact residues improving HLA-A2 binding [36]. Guichard and other groups have being using D-amino acids that produce retro-inverso analogues that have the direction of the peptide bonds reversed. Retro-inverso peptides are not susceptible to proteolytic degradation by naturally occurring peptidases, and show significantly improved bioavailability [37]. *Quesnel et al.* introduced backbone modifications, aminomethylene (CH2-NH) surrogate into the same melanoma peptide MART-1_27–35_. Five analogs bound MHC more efficiently than the parent peptide and two were recognized by one melanoma-specific T cell clone [38]. Most recently *Douat-Casassus et al.* have modified the central amino acids of the melanoma MART-1_26–35_ peptide using nonpeptidic units in order to stimulate a stronger T-cell response. Two compounds had high affinity for the HLA molecule and stimulated several Melan-A/MART-1 specific T-cell clones [24].

In conclusion, the incorporation of appropriate non-natural chemical entities into peptide-based vaccines for cancer immunotherapy may improve biological activity and provide new information on peptide processing. Modification of the tumor antigenic peptides at the anchor positions can enhance MHC binding while modification of TcR contact residues can enhance CTL responses. In this paper we have shown the successful incorporation of non-natural amino acids into T cell epitopes in both the anchor and TcR binding positions. The incorporation of a fluorine in a TcR binding position has generated a peptide that elicits a better immune response than the native sequence, which was able to recognize and kill WT1+ cancer cells. Photo-reactive analogs were also capable of covalently attaching to the MHC carrier, but this modification did not enhance immunogenicity because the half-life of the peptide in the MHC was dominated by the catabolism of the complex and the level of covalent interaction was low. Thus chemical modifications may provide alternative pathways for the rational design of peptides with applications in anti-tumor specific immunotherapy and in vaccine development.

## Supporting Information

Figure S1 T2 stabilization assay using peptides derived from WT1J. The assay was conducted as described in Material and Methods, with each panel representing a different experiment. Sequences of the peptides are shown in Table 1. The Y axis shows the mean fluorescence or the binding Index, that is the ratio between the median fluorescence with the peptide tested divided by median fluorescence with irrelevant peptide. Mean fluorescence was used for time course studies as the indices became low at the later time points with loss of MHC. The X axis show the time-points of incubation of the peptide tested or the different concentrations of the peptide tested. 1 min refers to UV irradiation of the peptide for 1 min after adding it to the cells.
(5.71 MB TIF)

Figure S2 T2 stabilization assay using peptides derived from WT1B. The assay was conducted as described in Fig. 1, with each panel representing a different experiment. Sequences of the peptides are shown in Table 1.
(5.61 MB TIF)
