# Supplementary figures and images for: Non-Natural and Photo-Reactive Amino Acids as Biochemical Probes of Immune Function
Source: PLoS One. 2008 Dec 15;3(12):e3938. doi: 10.1371/journal.pone.0003938 (PMC2592539; doi:10.1371/journal.pone.0003938)

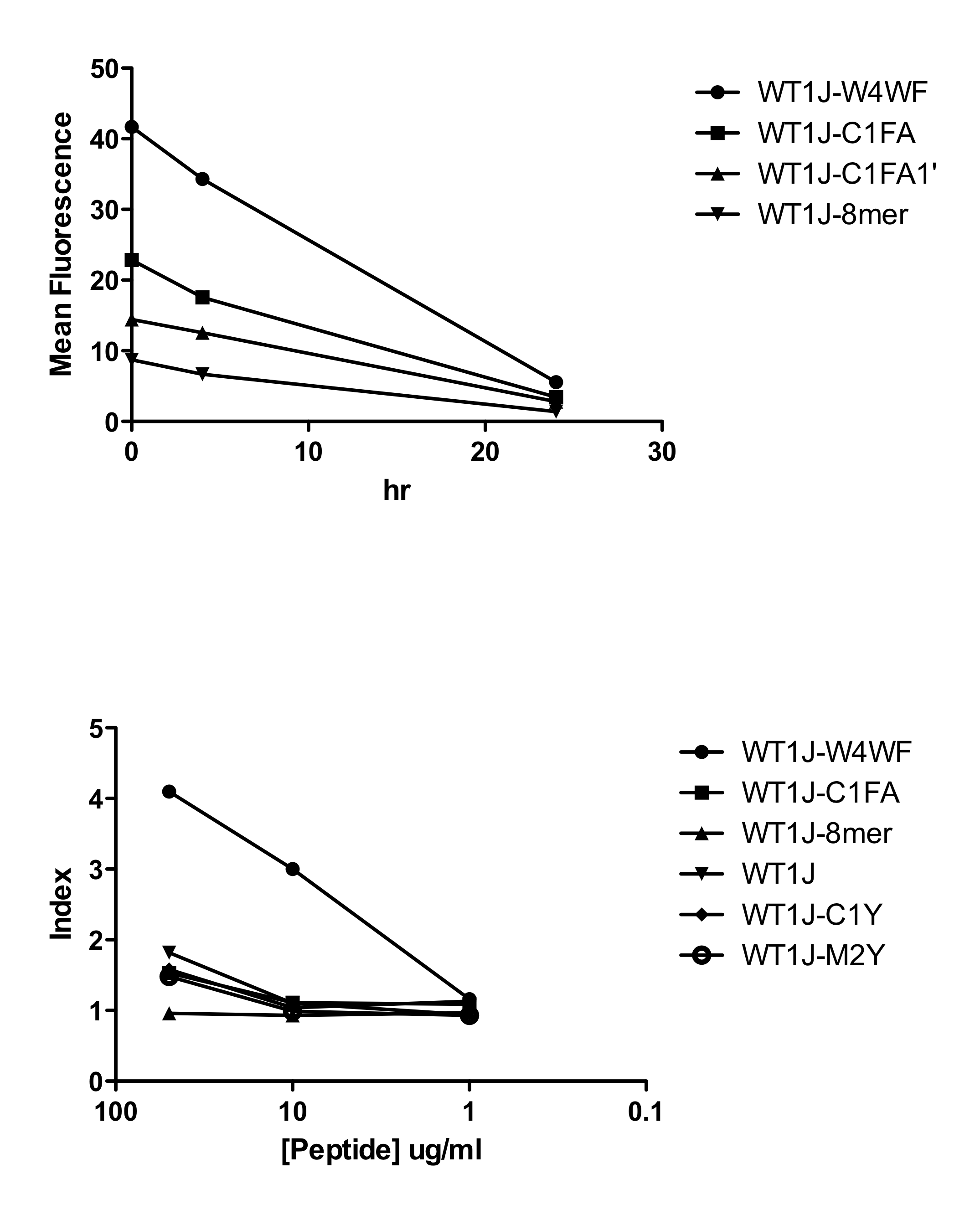

Supplement: Figure S1 — T2 stabilization assay using peptides derived from WT1J. The assay was conducted as described in Material and Methods, with each panel representing a different experiment. Sequences of the peptides are shown in Table 1. The Y axis shows the mean fluorescence or the binding Index, that is the ratio between the median fluorescence with the peptide tested divided by median fluorescence with irrelevant peptide. Mean fluorescence was used for time course studies as the indices became low at the later time points with loss of MHC. The X axis show the time-points of incubation of the peptide tested or the different concentrations of the peptide tested. 1 min refers to UV irradiation of the peptide for 1 min after adding it to the cells. (5.71 MB TIF) [file pone.0003938.s001.tif]

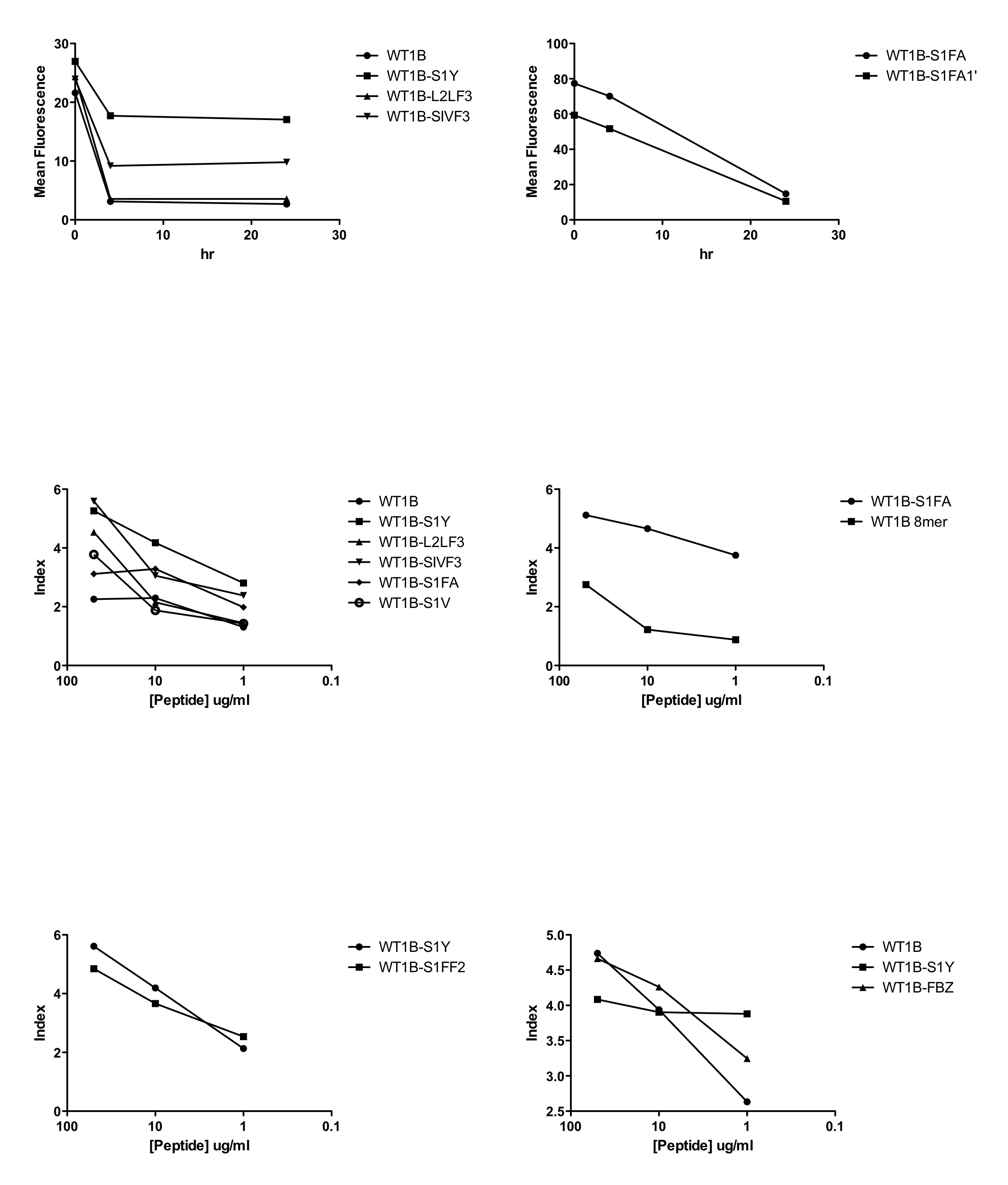

Supplement: Figure S2 — T2 stabilization assay using peptides derived from WT1B. The assay was conducted as described in Fig. 1, with each panel representing a different experiment. Sequences of the peptides are shown in Table 1. (5.61 MB TIF) [file pone.0003938.s002.tif]
